# Integrated histopathology and single-cell transcriptomics reveal distinct pathological features and foamy macrophage characteristics in spinal tuberculosis and brucellar spondylitis

**DOI:** 10.3389/fimmu.2026.1909083

**Published:** 2026-08-21

**Authors:** Qiang Liu, Hanhua Guo, Junxiao Liu, Zhengyong Tao, Long Ma, Zhangui Gu, Xuefeng Yue, Daihao Wei, Ningkui Niu, Jiandang Shi, Zongqiang Yang

**Affiliations:** 1 Department of Orthopedic, General Hospital of Ningxia Medical University, Yinchuan, Ningxia Hui Autonomous Region, China; 2 The First Clinical College of Ningxia Medical University, Yinchuan, Ningxia Hui Autonomous Region, China; 3 Research Center for Prevention and Control of Bone and Joint Tuberculosis, General Hospital of Ningxia Medical University, Ningxia, China

**Keywords:** brucellar spondylitis, cell-cell communication, foam cell, lipid metabolism, macrophage, single-cell RNA sequencing, spinal tuberculosis

## Abstract

**Background:**

Spinal tuberculosis (STB) and brucellar spondylitis (BS) show substantial overlap in clinical and imaging manifestations, but their local immunopathological programs and corresponding cellular states have not been systematically compared. This study integrated histopathology and single-cell transcriptomics to compare lesion-level differences between STB and BS and to characterize macrophage states associated with the foamy cell reaction.

**Methods:**

This retrospective case-control study included 80 STB cases and 80 BS cases treated between January 2020 and December 2024. H&E-stained lesion tissues from the full surgical cohort were reviewed for comparative histopathology. Fresh lesion nucleus pulposus tissue from six representative cases (STB = 3, BS = 3) underwent scRNA-seq re-analysis with cell-type annotation, myeloid subclustering, functional module scoring, GSVA, and CellChat analysis. Dual immunofluorescence for CD68 paired with CYP27A1, PLIN2, or PPARγ was used as tissue-level validation.

The scRNA-seq workflow focused on the monocyte-macrophage lineage to evaluate lipid-remodelled states associated with the foamy cell reaction. Tissue-level immunofluorescence and supplementary statistical summaries were interpreted as orthogonal support for the transcriptomic findings rather than as stand-alone diagnostic criteria.

**Results:**

Histopathological comparison showed that STB and BS were both dominated by chronic inflammation but exhibited distinct lesion patterns. STB was characterized mainly by tuberculous nodules, caseous necrosis, Langhans giant cells, and granulomatous inflammation, whereas BS more often showed plasma cell infiltration, foamy cell reaction, eosinophil infiltration, and fibrotic repair. These between-group differences were statistically significant (*P* < 0.05 or *P* < 0.01, as appropriate).

scRNA-seq yielded 67,274 high-quality cells and identified nine major cell types. Re-clustering of the Mono_Macrophage_DC lineage identified 15 subclusters, among which Macro4 was relatively enriched in BS. Rather than showing the highest value for every broad foam-cell-related metric, Macro4 was supported by convergent evidence including relative enrichment in BS, coordinated expression of PPARG, APOE, APOC1, PLIN2, and CYP27A1, and higher lipid-droplet-formation and cholesterol-efflux-related programs in BS_Macro4.

Dual immunofluorescence showed higher CD68-normalized overlap fractions for CYP27A1, PLIN2, and PPARγ in BS lesion tissues than in STB lesion tissues (*P* < 0.05). CellChat analysis further suggested that Macro4 contributed relatively more to the HGF-MET signalling context in BS, although these ligand-receptor relationships should be interpreted as transcriptome-inferred candidate interactions.

**Conclusions:**

STB and BS exhibit distinct local immunopathological programs. STB is characterized by a granulomatous-necrotizing inflammatory pattern, whereas BS more often shows a chronic inflammatory and tissue-repair-associated lesion background with a more prominent foamy cell reaction. Macro4 is best interpreted as a BS-associated lipid-remodelled macrophage state supported by convergent histological and transcriptomic evidence. These findings provide a lesion-level cellular framework for further pathological and mechanistic study rather than a validated stand-alone diagnostic cutoff.

## 1 Introduction

STB and BS are two major forms of chronic infectious spinal disease, with considerable overlap in clinical presentation, laboratory findings, and imaging features. Both conditions may present with chronic back pain, fever, night sweats, elevated inflammatory markers, and vertebral-disc destruction, making differential diagnosis challenging without definitive microbiological evidence (1). Although pathogen-based testing, including culture, PCR, and molecular assays, remains the diagnostic gold standard, its clinical utility may be limited by suboptimal pathogen detection, difficulty obtaining representative tissue, and prior antibiotic exposure, which can lead to culture negativity or reduced molecular sensitivity (2, 3). Molecular assays such as Xpert MTB/RIF can improve rapid pathogen detection in selected patients (4), and imaging features, machine-learning models, and serological or molecular biomarkers have also been explored to assist differentiation (5–7). Diagnostic models have also been developed to distinguish tuberculous spondylitis from brucellar spondylitis (8). However, these approaches are mainly adjunctive, and when lesion tissue is available, tissue-based analysis remains important for understanding the local disease microenvironment and immunopathological mechanisms.

From a histological perspective, STB and BS show both distinct and overlapping patterns of inflammation and tissue destruction. Typical STB lesions are characterized by granulomatous inflammation, caseous necrosis, tuberculous nodules, and Langhans giant cells, whereas BS lesions more commonly exhibit chronic inflammatory cell infiltration, plasma cell enrichment, tissue repair responses, and diverse immune-cell involvement (9). Recent studies have suggested that reliance on imaging or laboratory indices alone may result in diagnostic misclassification, whereas integration of histopathology with molecular testing can improve diagnostic accuracy (2, 4). Histopathology combined with targeted molecular testing has also shown value for spinal tuberculosis diagnosis (10). Nevertheless, systematic analyses of the local immune microenvironment in STB and BS remain scarce, particularly with respect to defined macrophage states and lipid metabolic remodelling.

Macrophages are key immune effector cells within chronic infectious lesion microenvironments and participate in pathogen recognition, phagocytosis, inflammatory regulation, and tissue repair. Their functional states are highly plastic and may be shaped by microenvironmental cues and persistent pathogen exposure, leading to polarization and metabolic reprogramming (11). Lipid metabolic remodelling and cholesterol efflux are important adaptive programs in macrophages during chronic infection and inflammation and may influence local immune responses, tissue remodelling, and disease progression (12). Lipid droplets are functionally active organelles in monocyte-macrophage biology and inflammatory regulation (13, 14), and lipid-droplet-associated pathways can regulate lipophagy and cholesterol efflux in macrophage foam cells (15). Foam cells represent a prototypical lipid-remodelled macrophage population and have been described in both atherosclerosis and chronic infectious lesions (16). However, their distribution, functional state, and pathological significance within STB and BS lesion microenvironments have not been systematically defined.

scRNA-seq provides a high-resolution approach for dissecting immune heterogeneity within complex lesion tissues. This technology enables the identification of disease-associated immune cell subsets, functional states, and transcriptional regulatory programs, thereby offering a molecular basis for understanding local pathological divergence (17). In tuberculosis infection, published single-cell studies have demonstrated heterogeneous macrophage lineages and functional states within infected lung tissue and necrotizing granulomatous lesions (11, 17). Integrating histopathology with single-cell analysis may help clarify the cellular origins and functional characteristics underlying the foamy cell response in BS lesions and its relationship with tissue injury, providing a more detailed perspective on immunopathological differences between STB and BS.

Against this background, the present study integrated histopathology with single-cell transcriptomics to compare lesion tissues from patients with STB and BS. Particular emphasis was placed on monocyte-macrophage heterogeneity, lipid-remodelled states associated with foamy macrophages, functional phenotypes, and candidate intercellular communication patterns. The novelty of this study lies in connecting conventional lesion morphology with spinal lesion-derived macrophage states and candidate communication programs in two clinically confusable spinal infections. By integrating tissue morphology, cellular composition, and molecular function, this study aimed to define the cellular basis of local immune microenvironmental differences between STB and BS and to provide evidence for pathological differentiation and future mechanistic investigation of these two infectious spinal diseases.

## 2 Materials and methods

### 2.1 Inclusion and exclusion criteria

Inclusion criteria: (1) definitive diagnosis based on medical history, clinical manifestations, laboratory examinations, imaging findings, and bacteriological or molecular evidence; for STB this included positive culture, Xpert MTB/RIF, mNGS/tNGS, or acid-fast-positive histopathology, whereas BS required Brucella culture or mNGS/tNGS evidence together with compatible serological and/or blood-culture findings; (2) clear surgical indication; (3) sufficient lesion tissue for pathological assessment; and (4) acceptable compliance with perioperative management.

Exclusion criteria: (1) tuberculosis or brucellosis involving other organ systems; (2) systemic immune disease; (3) poor general condition due to other major underlying disease; or (4) contraindication to surgery.

### 2.2 Clinical data and sample collection

#### Clinical data

This retrospective case-control study included 80 patients with STB and 80 patients with BS treated in the Department of Orthopaedics, General Hospital of Ningxia Medical University, between January 2020 and December 2024. H&E-stained lesion sections from all 160 cases were used for comparative histopathology. Fresh lesion nucleus pulposus tissue from six representative patients (STB = 3, BS = 3) was used for scRNA-seq re-analysis. All participants provided written informed consent, and the study was approved by the institutional ethics committee. The clinical characteristics of the sequencing cohort are summarized in **Supplementary Table 1**.

#### Sample collection

Patients with STB received 2–4 weeks of standard anti-tuberculosis therapy before surgery, whereas patients with BS received 2–3 weeks of anti-brucellosis therapy before surgery. Liver and renal function were monitored during preoperative treatment. Nutritional status and operative tolerance were assessed before surgery in both groups.

All patients underwent surgery under general anaesthesia. Depending on lesion location and stability requirements, posterior, anterior, or combined posterior-anterior debridement was performed, followed by intervertebral bone graft fusion at the affected level and instrumental internal fixation when indicated. Tissue specimens for routine pathology and sequencing were collected during lesion debridement by the operating team in conjunction with an experienced pathologist.

**Figure 1:**
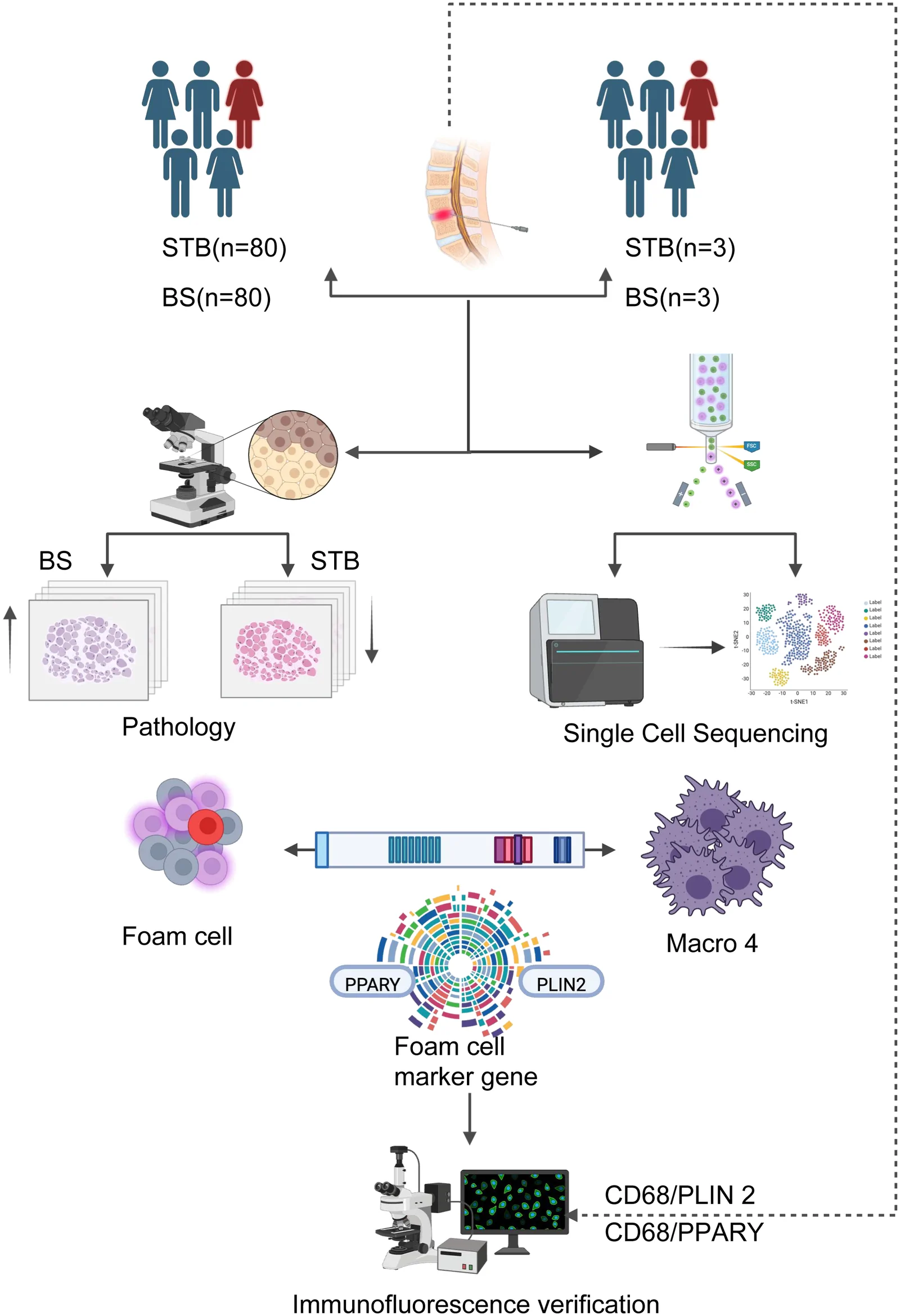
Study design and overall analytical workflow. Schematic overview of the study design. The full surgical cohort (STB, n = 80; BS, n = 80) was used for comparative histopathology, whereas only six fresh lesion samples (STB = 3, BS = 3) underwent centralized scRNA-seq. Downstream analyses included quality control, clustering, cell-type annotation, myeloid subclustering, functional scoring, GSVA, candidate cell-cell communication analysis, and tissue-level dual immunofluorescence validation.

Because scRNA-seq requires high-quality viable cells, only six fresh lesion samples (STB = 3, BS = 3) that were processed immediately after intraoperative collection were submitted for centralized sequencing. These specimens were washed with precooled medium, placed in preservation solution at 4 °C, and transported under cold-chain conditions to OE Biotech Co., Ltd. (Shanghai, China), with transport completed within 12 hours. The remaining surgical specimens were used for routine histopathology rather than sequencing. The overall study design and analytical workflow are shown in **Figure 1**.

### 2.3 Histopathological examination of tissue specimens by haematoxylin and eosin staining

**Figure 2:**
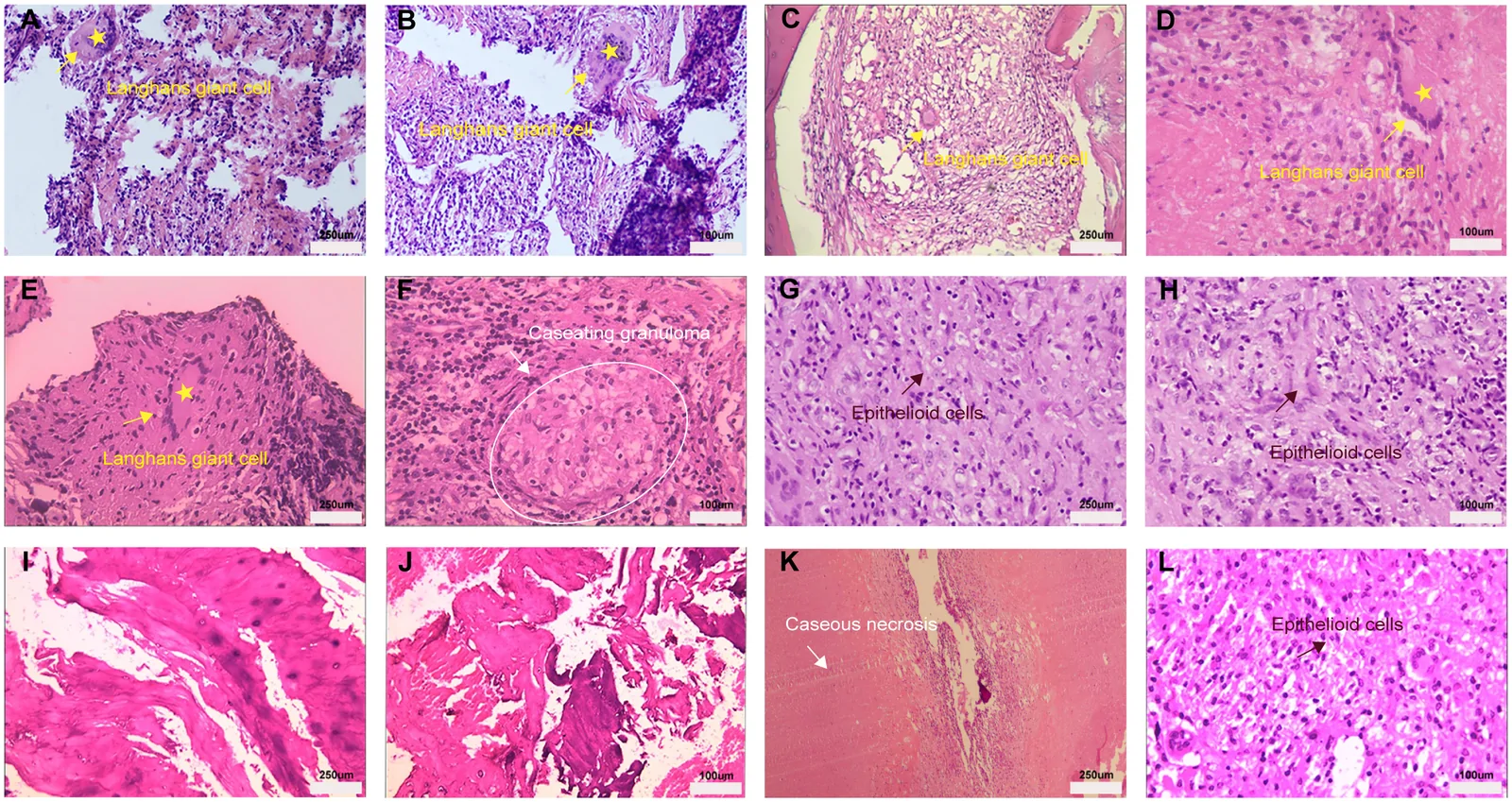
Histopathological features of STB. Representative H&E images from STB lesions. Panels **(A–L)** were selected from different STB patients to illustrate recurrent lesion-level pathological patterns observed in the cohort; matched uninvolved tissue was not routinely collected during surgical debridement. The annotations indicate representative histopathological features: yellow arrows and asterisks indicate multinucleated/Langhans-type giant cells; white arrows and circles indicate caseous granulomas; and brown-red arrows indicate epithelioid cells. The annotations highlight representative features rather than all pathological changes within each lesion. **(A)** Fragmented bone and fibrocartilaginous tissue with focal caseous necrosis-like areas. **(B)** Necrotic foci surrounded by epithelioid cells, inflammatory cells, and multinucleated/Langhans-type giant cells. **(C)** Fibrous tissue proliferation with inflammatory cell infiltration and focal necrotic areas. **(D)** Granuloma-like reaction composed of epithelioid cells, scattered multinucleated giant cells, inflammatory cells, necrosis, and fibrosis. **(E)** Cartilage and bone tissue with extensive inflammatory cell infiltration and focal coagulative necrosis. **(F)** Granulomatous inflammatory reaction composed mainly of epithelioid cells and inflammatory cells. **(G)** Fragmented bone tissue and proliferative fibrous tissue with focal necrotic lesions. **(H)** Granulomatous inflammatory reaction composed of lymphocytes, epithelioid cells, and multinucleated giant cells. **(I)** Proliferative cartilage tissue with necrotic tissue admixed with a small amount of sequestrum. **(J)** Inflammatory reaction surrounding necrotic foci, composed of lymphocytes, epithelioid cells, and a small number of multinucleated giant cells. **(K)** Bone, cartilage, and fibrous tissue with relatively well-demarcated focal caseous necrosis. **(L)** Granulomatous inflammatory reaction composed of epithelioid cells, lymphocytes, and multinucleated giant cells.

**Figure 3:**
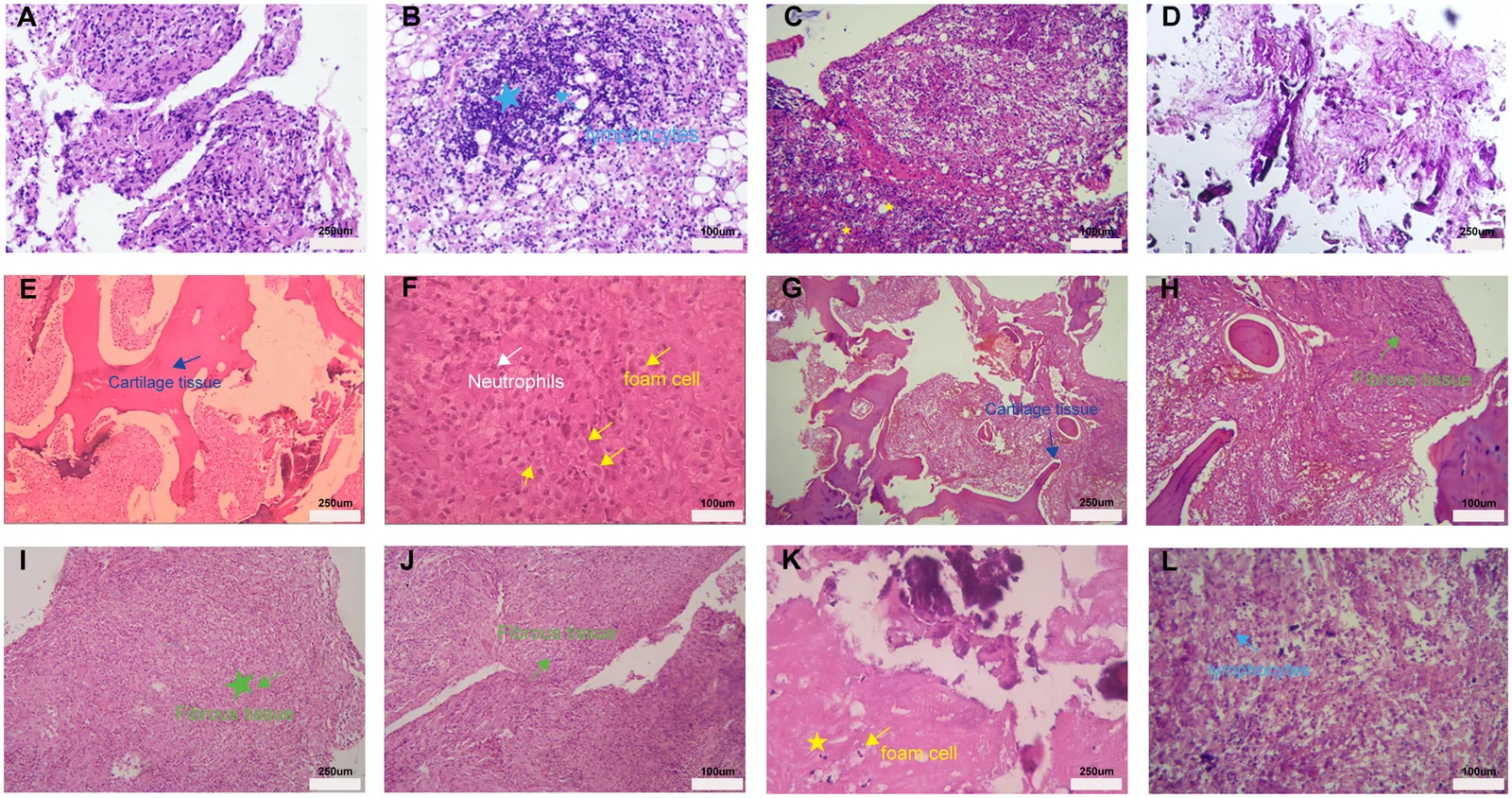
Histopathological features of BS. Representative H&E images from BS lesions. Panels **(A–L)** were selected from different BS patients to illustrate recurrent lesion-level patterns within the cohort; matched uninvolved tissue was not routinely collected during surgical debridement. The annotations indicate representative histopathological features: sky-blue arrows and asterisks indicate lymphocytes; yellow arrows and asterisks indicate foamy cells; dark-blue arrows indicate cartilage tissue; green arrows indicate fibrous tissue; and white arrows indicate neutrophils. The annotations highlight representative features rather than all pathological changes within each lesion. **(A)** Predominant fibrous tissue proliferation with abundant lymphocyte infiltration and scattered plasma cells in the stroma. **(B)** Predominant lymphocyte-rich inflammatory infiltration accompanied by histiocytic and foamy cell reaction. **(C)** Diffuse inflammatory cell infiltration, mainly composed of lymphocytes and plasma cells. **(D)** Fibrous tissue proliferation with partial necrotic changes. **(E)** Coagulative necrotic foci. **(F)** Mixed inflammatory infiltration composed of abundant lymphocytes, foamy cells, eosinophils, and neutrophils. **(G)** Bone and cartilage structures with diffuse inflammatory lesions. **(H)** Abundant lymphocyte and plasma cell infiltration accompanied by histiocytic and foamy cell reactions. **(I)** Marked fibrous tissue proliferation. **(J)** Chronic inflammatory changes dominated by histiocytic and foamy cell reaction, accompanied by marked lymphocyte infiltration. **(K)** Lesions mainly composed of proliferative cartilage and a small amount of bone tissue, with focal fibrinoid necrosis and haemorrhage. **(L)** Lesions characterized by massive neutrophil infiltration and vascular proliferation.

All 160 surgical lesion specimens were fixed in 4% formalin within 30 minutes after removal and processed by routine decalcification, dehydration, paraffin embedding, sectioning, and H&E staining. For each case, at least three representative fixed H&E sections from the lesion area were reviewed by an experienced chief pathologist, and histopathological features were recorded and scored at the case level rather than at the image level. Several typical lesion sections from different patients were then selected as representative images for **Figures 2**, **3**. Because surgery was directed to infected tissue, adjacent uninvolved tissue was not routinely collected as a matched control.

The raw sequencing data generated in this study have been deposited in the Genome Sequence Archive for Human (GSA-Human) under accession numbers HRA017497 (single-cell RNA-seq) and HRA017547 (bulk RNA-seq).

### 2.4 scRNA-seq and data analysis

1. Single-cell dataset and preprocessing. Tissue dissociation, viable single-cell suspension preparation, library construction, and sequencing were performed as described in our previous study based on the same lesion-derived single-cell dataset and sequencing workflow (18). Only suspensions with >90% viability were used for library preparation. Raw sequencing data from six lesion samples (STB = 3, BS = 3) were processed with Cell Ranger using STAR-based alignment, and downstream analyses were conducted within the Seurat framework (19). The analytical workflow followed widely used single-cell integration and annotation principles implemented in the Seurat ecosystem (20). Cells were excluded if they had fewer than 200 detected genes, fewer than 1,000 UMIs, log10(genes per UMI) < 0.7, mitochondrial UMI content >10%, or haemoglobin gene content >5%. Residual doublets were further removed with DoubletFinder.
2. Cell-type annotation and functional module scoring. To minimize inter-sample batch effects, cross-sample integration was performed with the mutual nearest neighbours (MNN) algorithm, followed by principal-component analysis, UMAP visualization, and graph-based shared nearest neighbour clustering with resolution = 0.5. Major cell types were annotated using a combined strategy based on canonical markers, cluster-level differential expression, and the overall expression pattern of each cluster rather than any single non-canonical marker alone. **Supplementary Table 3** lists representative marker genes for the major cell types, and **Supplementary Table 8** summarizes the tissue-validation marker rationale and ImageJ-based CD68-normalized overlap quantification workflow. Seurat AddModuleScore was used to calculate M1/M2 polarization scores and additional functional modules, including lipid-droplet-formation- and cholesterol-efflux-related signatures.
3. Gene set variation analysis. GSVA was performed using the GSVA R package (v1.46.0) on log2-transformed normalized expression matrices to compare pathway activity across disease groups and macrophage subsets (21).
4. Differential expression and transcription-factor-related analysis. Differentially expressed genes and transcription factors between predefined groups or subclusters were identified with Seurat FindMarkers using the presto method (test.use = “presto”) (19). Genes with Benjamini-Hochberg-adjusted *P* < 0.05 and fold change > 1.5 were considered statistically significant. Heatmaps were generated with ComplexHeatmap to summarize cluster-level and disease-stratified regulatory patterns (22).
5. Cell-cell communication analysis. CellChat (v2.1.2) was used to infer candidate intercellular communication networks separately in STB and BS (23). Communication probabilities were filtered with min.cells = 10. Macro4-related interaction strength, interaction number, and pathway-level probability differences were summarized in **Supplementary Table 7**.

### 2.5 Dual immunofluorescence staining

**Figure 4:**
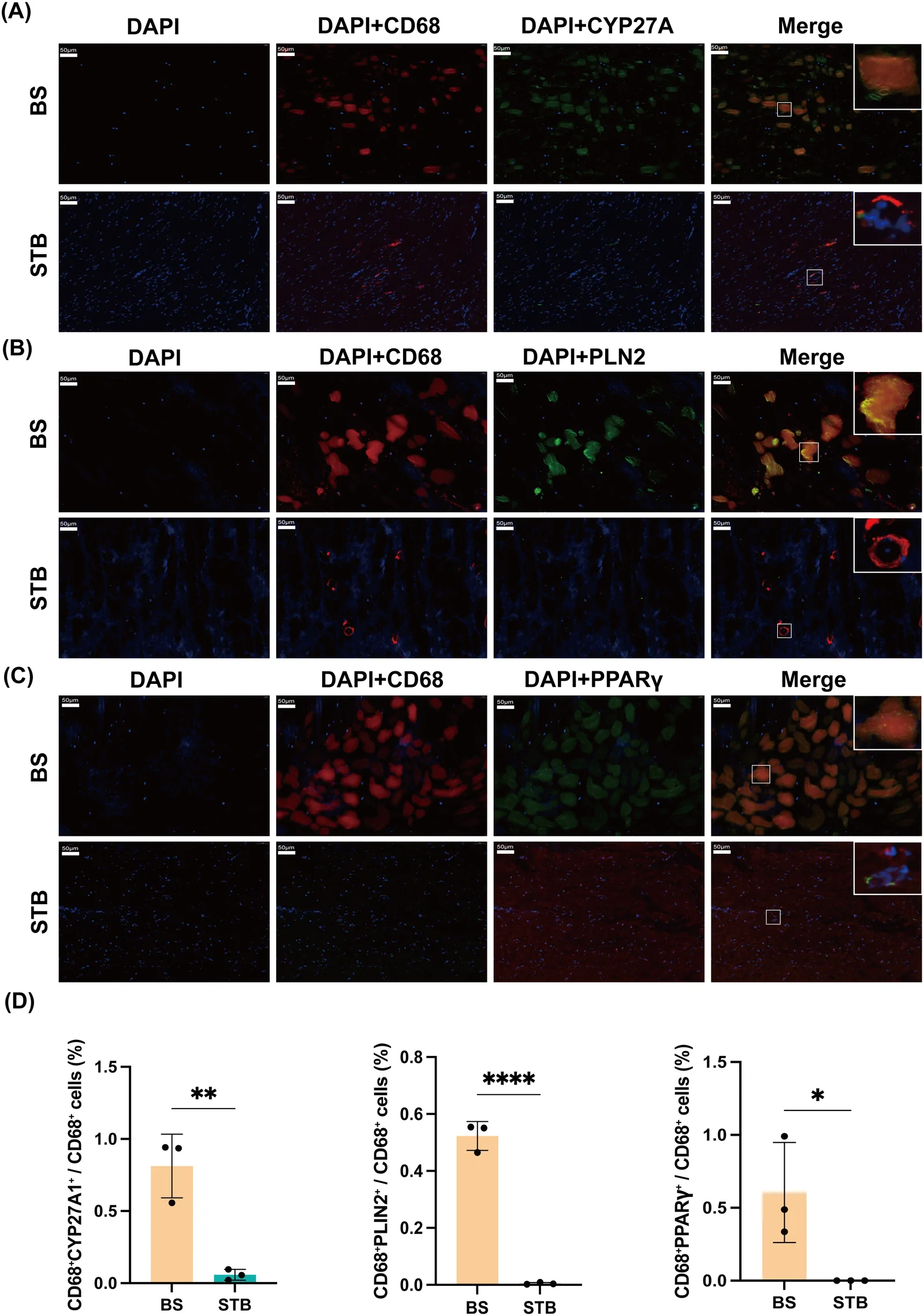
Dual immunofluorescence validation of lipid-remodelled macrophage features in BS lesions. **(A–C)** Representative dual immunofluorescence images from BS and STB lesion tissues showing CD68 paired with CYP27A1, PLIN2, or PPARγ, respectively. Local enlarged merge views are shown to improve visualization of double-positive signals. **(D)** ImageJ-based quantification of the CD68-normalized overlap fraction under a unified thresholding strategy, calculated as the overlap area between CD68 and the target marker divided by the total CD68-positive area (Overlap/CD68). Each group included three cases, and three representative 20x lesion fields were analysed per case. Because the analysis was performed on lesion tissue rather than matched uninvolved control tissue, these results should be interpreted as orthogonal tissue-level support for lipid-remodelled macrophage features rather than direct one-to-one identification of the scRNA-seq-defined Macro4 cluster. Data are presented as mean ± SEM. * P < 0.05; ** P < 0.01; **** P < 0.0001.

Paraffin-embedded lesion sections were deparaffinized, rehydrated, subjected to antigen retrieval, and incubated with primary antibodies against CD68 together with CYP27A1, PLIN2, or PPARγ, followed by species-matched fluorescent secondary antibodies and DAPI counterstaining. Images from each staining set were acquired under matched exposure settings. For **Figure 4D**, each group included three cases, and three representative 20x lesion fields were analysed per case. ImageJ was used for unified thresholding of the CD68 and target-marker channels. The quantitative endpoint was the CD68-normalized overlap fraction, calculated as the area positive for both CD68 and the target marker divided by the total CD68-positive area (Overlap/CD68). This endpoint indicates the proportion of the CD68-positive macrophage area that co-expressed CYP27A1, PLIN2, or PPARγ. Because no matched uninvolved tissue was routinely collected during surgical debridement, the immunofluorescence comparison focused on STB versus BS lesion tissue.

### 2.6 Statistical analysis

Statistical analyses were performed using R software and GraphPad Prism (v9.0), incorporating the relevant bioinformatics and statistical packages. Pathological categorical data are presented as numbers and percentages and were compared using the chi-square test or Fisher’s exact test, as appropriate. Continuous variables from immunofluorescence or summary statistics are presented as the mean ± SEM or as the median with interquartile range (IQR), depending on data distribution, and were compared using unpaired, two-tailed Student’s t-tests or non-parametric alternatives, as appropriate.

Unless otherwise stated, single-cell module scores and differential-expression analyses were assessed using two-sided Wilcoxon rank-sum tests. Correlations were evaluated with Spearman’s analysis. Statistical significance was defined as follows: *P* < 0.0001 (****), *P* < 0.001 (***), *P* < 0.01 (**), *P* < 0.05 (*), and ns, not significant (*P* > 0.05). Exact tests and summary outputs corresponding to the main histopathology, module-score, and CellChat analyses are provided in the revised tables and **Supplementary Files**.

## 3 Results

### 3.1 Histopathological changes in STB under light microscopy

Pathologically, acute inflammation was characterised by neutrophils, eosinophils, necrosis, and abscess formation, whereas chronic inflammation was characterised by lymphocytes, plasma cells, fibrous tissue, mononuclear cells, hyaline degeneration, vascular proliferation, and other forms of tissue proliferation. Other findings included vascular dilatation, haemorrhage, granulomas, and multinucleated giant cells. Special microscopic findings included foamy cell reaction, histiocytic reaction, and eosinophilic abscess foci.

Histopathological changes in HE-stained tissue specimens from 80 patients with STB under light microscopy were as follows: no cases showed acute inflammation alone, 56 cases showed chronic inflammation, and 24 cases showed acute exacerbation of chronic inflammation. Overall, the pathological changes in tissue specimens from patients with STB were mainly characterised by chronic inflammatory changes, including infiltration of lymphocytes, plasma cells, and mononuclear cells. Lymphocytes were present in 95.00% of cases, with +++ infiltration observed in 20.00%; plasma cells were present in 57.50% of cases, with +++ infiltration observed in 6.25%; and mononuclear cells were present in 53.75% of cases, with +++ infiltration observed in 26.25%. Fibrous tissue proliferation was observed in 86.25% of cases, with +++ proliferation in 7.50%; eosinophils were observed in 20.00%; histiocytic reaction was observed in 77.50%, with +++ reaction in 10.00%; foamy cell reaction was observed in 22.50%; granulomatous inflammation was observed in 38.75%; and granulation tissue proliferation was observed in 25.00%. Typical pathological features of STB included caseous necrosis in 81.25% of cases, Langhans giant cells in 66.25%, and tuberculous nodules in 41.25%.

Among the 56 STB cases dominated by chronic inflammation alone, the microscopic findings were mainly characterised by infiltration of lymphocytes, plasma cells, and mononuclear cells. Histiocytic reaction was observed in 71.43% of cases, foamy cells in 14.29%, granulomatous inflammation in 39.29%, Langhans giant cells in 75.00%, and granulation tissue in 10.71%. Among the 24 STB cases with chronic inflammation accompanied by acute inflammation, the microscopic pathological findings were as follows: necrosis was observed in 91.67% of cases, eosinophils in 29.17%, abscess formation in 70.83%, histiocytic reaction in 91.67%, foamy cells in 41.67%, granulomatous inflammation in 37.50%, Langhans giant cells in 45.83%, and granulation tissue in 58.33% (**Supplementary Table 2**; **Figure 2**).

### 3.2 Histopathological changes in BS under light microscopy

Histopathological changes in HE-stained tissue specimens from 80 patients with BS under light microscopy were as follows: no cases showed acute inflammation alone, 28 cases showed chronic inflammation, and 52 cases showed acute exacerbation of chronic inflammation. Overall, the pathological changes in tissue specimens from patients with BS were mainly characterised by chronic inflammatory changes, including infiltration of lymphocytes, plasma cells, and mononuclear cells. Lymphocytes were present in 98.75% of cases, with +++ infiltration observed in 73.75%; plasma cells were present in 98.75% of cases, with +++ infiltration observed in 60.00%; mononuclear cells were present in 91.25% of cases, with +++ infiltration observed in 16.25%; fibrous tissue proliferation was observed in 88.75% of cases, with +++ proliferation in 43.75%; eosinophils were observed in 55.50%; histiocytic reaction was observed in 68.75%, with +++ reaction in 18.75%; foamy cell reaction was observed in 41.25%; granulomatous inflammation was observed in 8.75%; multinucleated giant cells were observed in 7.50%; and granulation tissue proliferation was observed in 35.00%.

Among the 28 BS cases dominated by chronic inflammation alone, the microscopic findings were mainly characterised by infiltration of lymphocytes, plasma cells, and mononuclear cells. Histiocytic reaction was observed in 39.26% of cases, foamy cells in 17.86%, granulomatous inflammation in 10.71%, multinucleated giant cells in 10.71%, and granulation tissue in 14.30%. Among the 52 BS cases with chronic inflammation accompanied by acute inflammation, the microscopic pathological findings were as follows: necrosis was observed in 78.84% of cases, eosinophils in 84.62%, abscess formation in 75.00%, histiocytic reaction in 78.84%, foamy cells in 48.08%, granulomatous inflammation in 7.69%, multinucleated giant cells in 5.77%, and granulation tissue in 46.15% (**Supplementary Table 2**; **Figure 3**).

### 3.3 Comparison of histopathological changes between STB and BS under light microscopy

The typical pathological features of STB included tuberculous nodules, caseous necrosis, Langhans giant cells, and granulomatous inflammation, which accounted for 41.25%, 81.25%, 66.25%, and 38.75%, respectively. In contrast, the proportions of tuberculous nodules, caseous necrosis, Langhans giant cells, and granulomatous inflammation in BS were 0%, 0%, 0%, and 8.75%, respectively. The differences between the two groups were statistically significant (*P* < 0.01). In the pathological features of STB, foamy cells accounted for 22.50%, eosinophils for 20.00%, and plasma cells for 57.50%. In BS, foamy cells accounted for 41.25%, eosinophils for 65.00%, and plasma cells for 98.75%. The differences between the two groups were statistically significant (*P* < 0.05), with significantly higher proportions in BS than in STB (**Table 1**; **Figures 2**, **3**).

**Table 1:** Comparison of histopathological changes between STB and BS under light microscopy.

Item	STB	BS	Chi-square	*P* value
Tuberculous nodules	33/80	0/80	41.575	<0.01
Caseous necrosis	65/80	0/80	109.474	<0.01
Langhans giant cells	53/80	0/80	79.252	<0.01
Granulomatous inflammation	31/80	7/80	19.879	<0.01
Foamy cell reaction	18/80	33/80	6.476	0.011
Histiocytic reaction	62/80	55/80	1.558	0.212
Eosinophil infiltration	16/80	52/80	33.146	<0.01
Lymphocyte infiltration	76/80	79/80	0.826	0.363
Plasma cell infiltration	46/80	79/80	39.826	<0.01
Mononuclear cell infiltration	43/80	73/80	28.213	<0.01
Granulation tissue proliferation	20/80	28/80	1.905	0.168

Microscopically, STB was characterized mainly by chronic inflammatory changes with granulomatous-necrotizing features, whereas BS more often showed chronic inflammation with tissue-repair-associated change and a more prominent foamy or histiocytic reaction. These histological patterns may provide adjunctive pathological clues when interpreted together with microbiological, serological, and clinical data, but they should not be considered a substitute for pathogen confirmation.

### 3.4 Clustering and characterisation of monocyte–macrophage populations in nucleus pulposus tissues from patients with STB and BS

**Figure 5:**
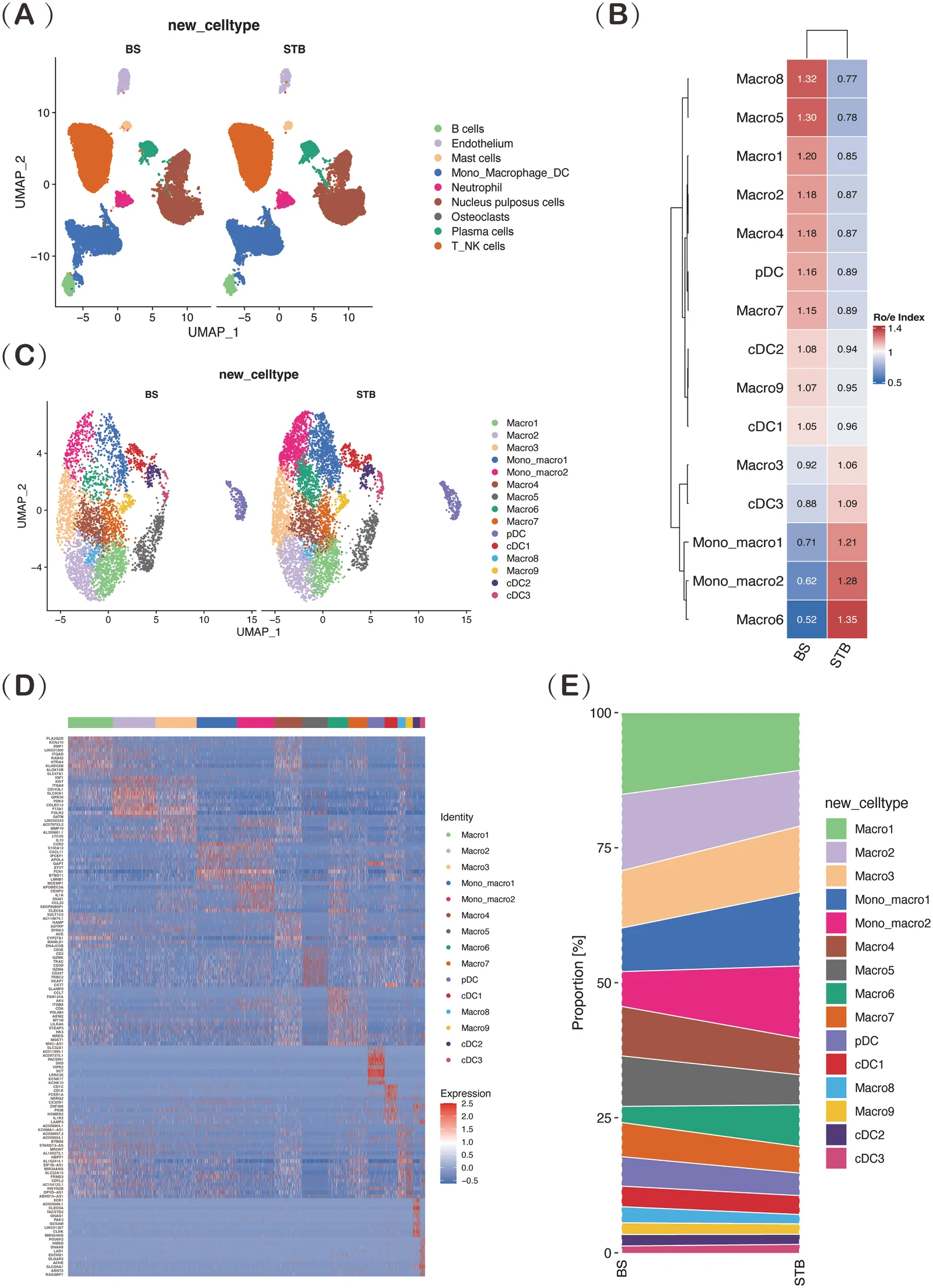
Single-cell atlas and monocyte–macrophage heterogeneity in STB and BS. **(A)** UMAP visualization of major cell types identified in nucleus pulposus tissues, including immune and stromal populations. **(B)** Re-clustering of the Mono_Macrophage_DC lineage reveals 15 subclusters, including nine macrophage subsets (Macro1–Macro9), two monocyte–macrophage transitional subsets (Mono_macro1–2), and four dendritic cell subsets (pDC, cDC1–cDC3). **(C)** Heatmap of canonical marker gene expression confirming cell-type identity across subclusters. **(D, E)** Relative enrichment (Ro/e) analysis showing differential distribution of subclusters between STB and BS, highlighting disease-specific myeloid remodeling.

In this study, single-cell RNA sequencing was performed on infectious nucleus pulposus tissues from six patients (STB = 3, BS = 3). After quality control and clustering analysis, a total of 67,274 high-quality cells were obtained. The major cell types were annotated according to canonical marker genes and previous single-cell studies, including B cells, endothelial cells, mast cells, Mono_Macrophage_DC cells, neutrophils, nucleus pulposus cells, osteoclasts, plasma cells, and T/NK cells (**Figure 5A**).

Mono_Macrophage_DC cells were then extracted for secondary clustering, which identified 15 subclusters, including nine macrophage subclusters (Macro1-Macro9), two monocyte-macrophage transitional subclusters (Mono_macro1 and Mono_macro2), and four dendritic cell subclusters (pDC, cDC1, cDC2, and cDC3) (**Figure 5B**; **Supplementary Table 3**). The marker gene expression profiles differed markedly among these subclusters, confirming their cellular identities and indicating substantial transcriptional heterogeneity within the monocyte-macrophage-dendritic cell lineage (**Figure 5C**).

To assess the compositional differences of these subclusters under different disease conditions, relative enrichment analysis based on Ro/e showed that Macro6, Mono_macro1, Mono_macro2, Macro3, and cDC3 were relatively enriched in STB, whereas Macro8, Macro5, Macro1, Macro2, Macro4, Macro7, pDC, cDC1, and cDC2 were relatively enriched in BS (**Figures 5D, E**; **Supplementary Table 4**). These findings indicate that multiple functional macrophage subclusters are present in BS lesions, suggesting marked functional remodelling. In contrast, STB lesions were characterised by enrichment of monocyte-macrophage transitional subclusters and specific macrophage subclusters, consistent with the immunological features of granulomatous inflammation.

### 3.5 Identification and functional analysis of foamy macrophages in nucleus pulposus tissues from patients with STB and BS

**Figure 6:**
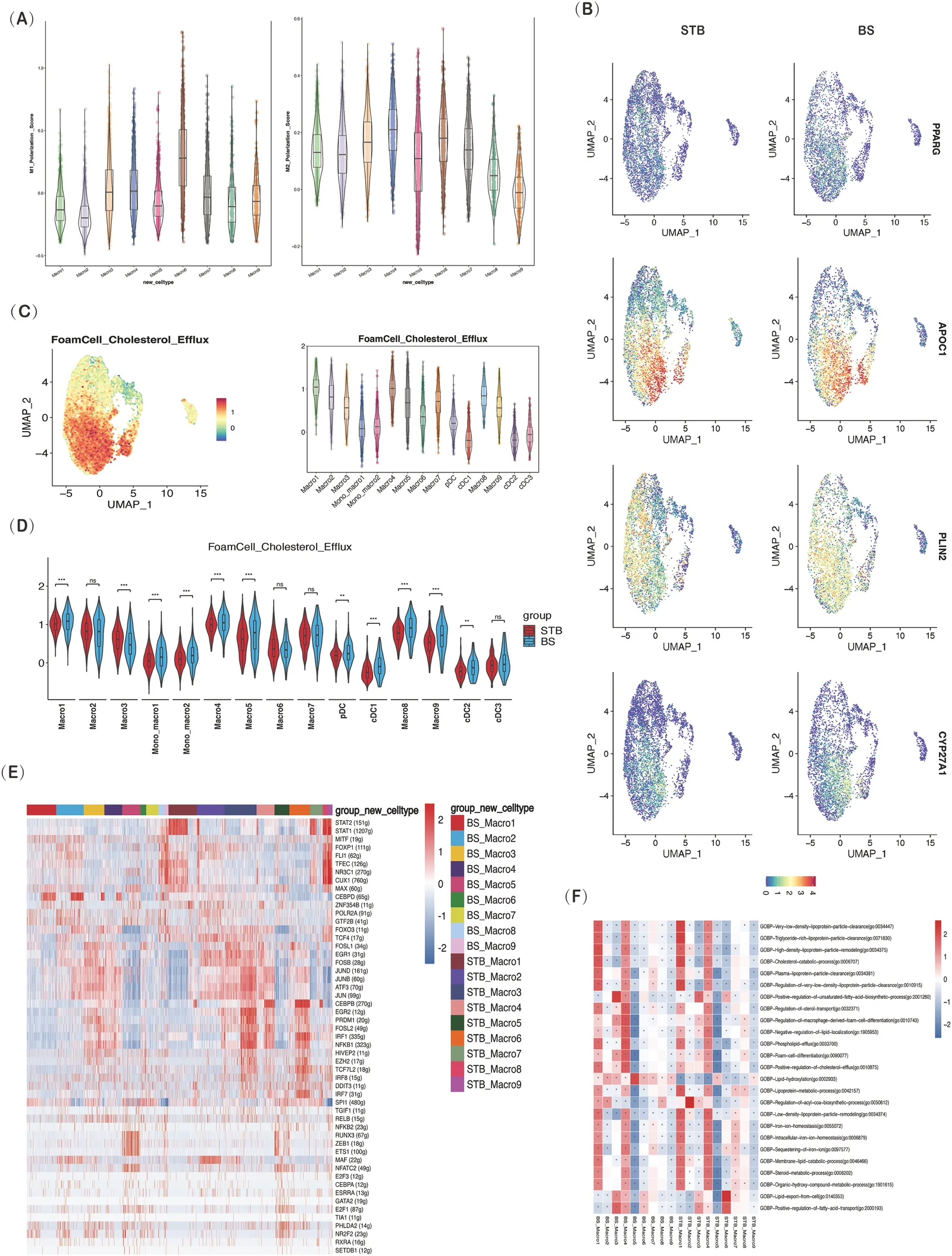
Identification and functional characterization of foamy macrophage subset Macro4. **(A)** M1/M2 module scores across myeloid subsets, showing functional heterogeneity rather than a single uniform polarization state. **(B)** UMAP feature plots showing enriched expression of lipid metabolism- and foam-cell-associated genes (PPARG, APOE, APOC1, PLIN2, CYP27A1) in the Macro4 region. **(C)** Distribution of the cholesterol-efflux-related module across myeloid cells. **(D)** Disease-stratified within-subset comparison of the FoamCell_Cholesterol_Efflux score, showing subset-specific differences between STB and BS, including higher activity in BS_Macro4 and a distinct trend in Macro3. **(E)** Transcription-factor/regulon activity heatmap summarizing disease-associated regulatory patterns. **(F)** GSVA heatmap showing enrichment of lipid handling, foam-cell differentiation, sterol transport, and related pathways in BS_Macro4. * P < 0.05; ** P < 0.01; *** P < 0.001; ns P > 0.05

To identify macrophage states related to the foamy cell reaction, we first examined lipid-handling and foam-cell-associated genes across myeloid subclusters. UMAP feature plots showed coordinated enrichment of PPARG, APOE, APOC1, PLIN2, and CYP27A1 in the Macro4 region, and relative enrichment analysis indicated that Macro4 was more enriched in BS than in STB. These findings support Macro4 as a BS-associated lipid-remodelled macrophage state rather than a diagnosis-defining cell population (**Figure 6B**; **Supplementary Tables 4**-**6**).

Module scoring showed substantial heterogeneity across myeloid subsets. M1/M2 scoring indicated that the myeloid subsets did not conform to a single uniform polarization pattern (**Figure 6A**). Importantly, the broad FoamCell_Cholesterol_Efflux score was not unique to Macro4, because several other macrophage subsets also showed relatively high values (**Figure 6C**). However, when disease groups were compared within each myeloid subset, BS_Macro4 showed higher cholesterol-efflux-related activity than STB_Macro4, whereas the generic foam-cell score alone did not explain the full difference. Macro3 also displayed a different disease-associated trend from several other subsets, underscoring that macrophage remodelling in STB and BS is heterogeneous rather than monotonic (**Figure 6D**; **Supplementary Table 5**).

Transcription-factor/regulon analysis further revealed distinct regulatory patterns across macrophage subsets. In BS-derived macrophages, MITF/TFEC, CEBPD, AP-1 family members, and IRF/NFKB-related factors were variably activated, suggesting coordinated programs related to macrophage activation, inflammatory adaptation, and lipid remodelling (**Figure 6E**). These patterns support the interpretation that BS_Macro4 represents a functionally remodelled state rather than a cell population defined by a single marker gene.

GSVA further showed that BS_Macro4 was enriched in pathways related to foam-cell differentiation, macrophage-derived foam-cell differentiation, cholesterol efflux, phospholipid efflux, lipoprotein particle clearance, sterol transport, and fatty-acid transport (**Figure 6F**; **Supplementary Table 6**), further supporting its lipid-remodelled phenotype.

Taken together, these data indicate that Macro4 is relatively enriched in BS and is characterized by coordinated lipid handling, cholesterol transport, and bacterial-response programs. We therefore interpret Macro4 as a lipid-remodelled macrophage state associated with the BS lesion microenvironment, while recognizing that no single score or marker alone is sufficient to define a disease-specific diagnostic cutoff.

### 3.6 Dual immunofluorescence validation provides histological evidence of foamy macrophages in BS

To validate, at the tissue level, the abnormal macrophage lipid metabolism identified by single-cell transcriptomic analysis, we further performed dual immunofluorescence staining for CD68 and lipid metabolism-related molecules, including CYP27A1, PLIN2 and PPARγ, to assess differences in the spatial distribution of foamy macrophages between STB and BS lesions (**Figure 4**).

Dual immunofluorescence showed higher CD68-normalized overlap fractions for CYP27A1, PLIN2, and PPARγ in BS lesion tissue than in STB lesion tissue (**Figures 4A–D**). These signals indicate that a larger proportion of the CD68-positive macrophage area in BS lesions co-expressed markers related to cholesterol handling, lipid-droplet biology, and lipid-metabolism-associated transcriptional activity.

The immunofluorescence data therefore support, but do not on their own prove, the presence of a lipid-remodelled macrophage population in BS lesions. Because dual staining was performed at the tissue level, these findings are interpreted as orthogonal support for the scRNA-seq-defined Macro4 program rather than direct one-to-one identification of Macro4 cells.

### 3.7 Analysis of Macro4-related intercellular communication and the HGF signalling pathway

**Figure 7:**
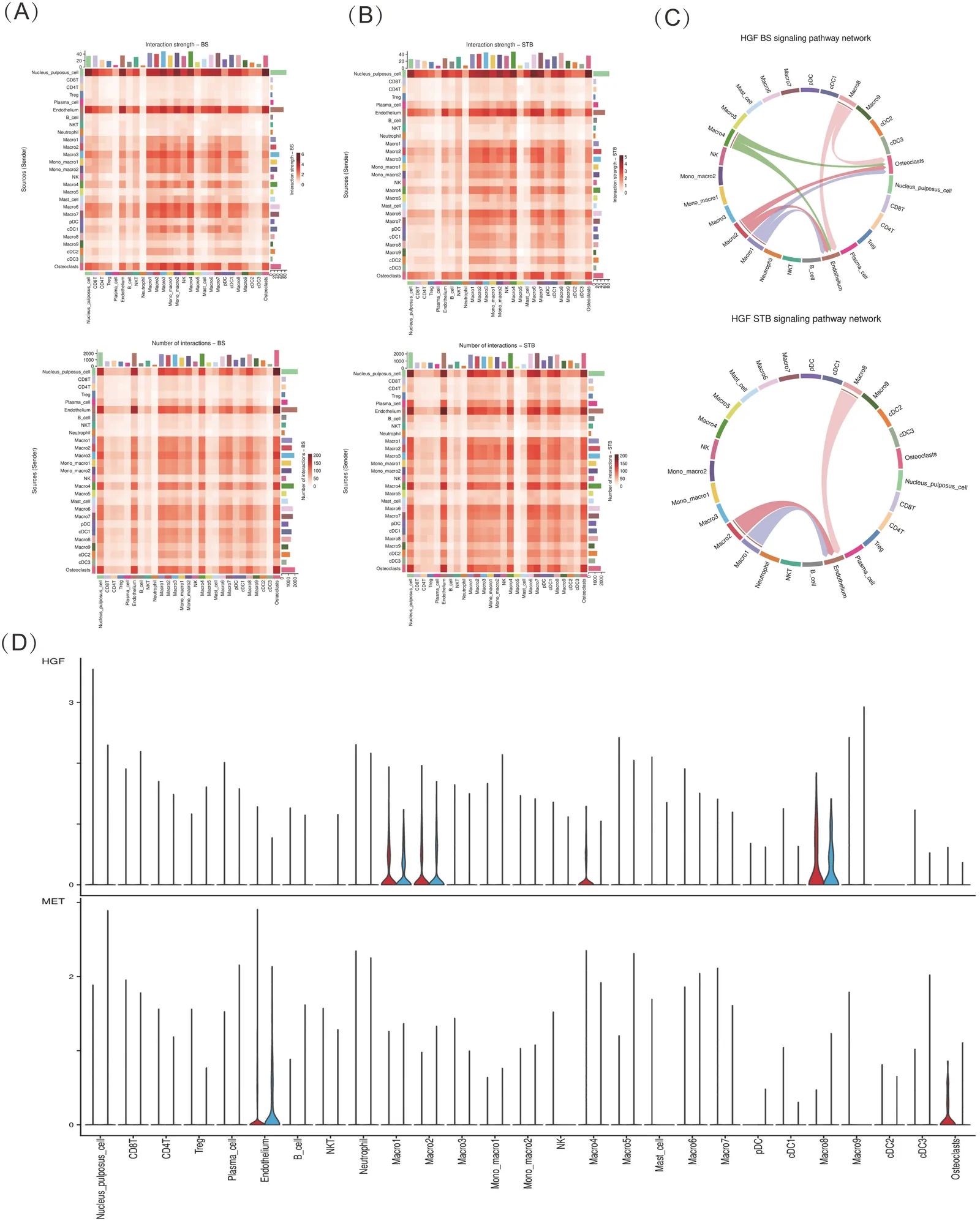
Macro4-associated intercellular communication and HGF–MET signalling network. **(A, B)** CellChat analysis revealing global cell–cell communication networks in STB and BS, identifying nucleus pulposus cells, endothelial cells, osteoclasts, and macrophages as major communication hubs. **(C)** Comparative HGF signalling network showing differential communication patterns between STB and BS. **(D)** Ligand–receptor expression analysis indicating macrophage-derived HGF and MET expression in endothelial cells, osteoclasts, and stromal cells. Overall, Macro4 exhibits enhanced participation in HGF-mediated signalling in BS, suggesting its role in macrophage–vascular–bone axis regulation and tissue remodelling.

To evaluate whether the lipid-remodelled Macro4 state might participate in lesion microenvironmental interactions, CellChat analyses were performed separately in STB and BS. Both diseases showed extensive predicted communication networks involving nucleus pulposus cells, endothelial cells, osteoclasts, and myeloid cells (**Figures 7A, B**).

Within these predicted networks, HGF signalling was active in both STB and BS, but the relative contribution of Macro4 was greater in BS. HGF was mainly expressed in macrophage-related subsets, whereas MET was concentrated in endothelial cells, osteoclasts, and some stromal populations, suggesting a candidate macrophage-to-endothelial/osteoclast communication axis (**Figures 7C, D**; **Supplementary Table 7**).

Because CellChat infers candidate ligand-receptor interactions from transcriptomic data, these findings should be interpreted as hypothesis-generating rather than causal proof. Nevertheless, they are consistent with the possibility that BS-associated lipid-remodelled macrophages contribute to tissue repair and bone microenvironment remodelling through candidate HGF-MET-related interactions, a pathway context relevant to MET receptor activation and macrophage-bone remodelling biology (24, 25).

## 4 Discussion

STB and BS are among the most clinically confusable pathogen-specific spinal infections. Their laboratory and imaging overlap makes tissue-level interpretation important when pathogen confirmation is delayed or incomplete. The present study integrated histopathology and lesion-derived scRNA-seq to compare these two diseases at both the morphological and cellular-state levels.

This study further demonstrates that, although STB and BS are both chronic infectious spinal diseases, their lesion tissues do not represent the same inflammatory response. Instead, they correspond to two distinct local immunopathological programmes. STB is dominated by a granulomatous-necrotising inflammatory pattern, whereas BS is more consistent with a chronic inflammation–tissue repair microenvironment. In STB lesions, tuberculous nodules, caseous necrosis, Langhans giant cells and granulomatous inflammation were markedly more frequent than in BS, indicating that STB lesions mainly exhibit a typical granulomatous-necrotising inflammatory pattern. Previous histopathological studies of spinal tuberculosis have also shown that caseous necrosis, abscess formation and granulation tissue formation are important histological features for pathological diagnosis and lesion assessment in STB (26). This pathological pattern reflects the process by which macrophages, epithelioid cells, multinucleated giant cells and lymphocytes jointly participate in granuloma formation under persistent stimulation by Mycobacterium tuberculosis. Single-cell and spatial transcriptomic studies have further shown that tuberculous lesions are not homogeneous inflammatory structures, but rather complex immune architectures composed of diverse macrophage states, lymphocyte states and tissue niches (11).

In contrast to STB, BS lesions lacked typical tuberculous nodules, caseous necrosis and Langhans giant cells, and showed a much lower proportion of granulomatous inflammation. Instead, plasma cell infiltration, mononuclear cell infiltration, eosinophil infiltration, foamy cell reaction and fibrous tissue proliferation were more prominent. These findings indicate that BS should not be interpreted simply as a weakened form of granulomatous inflammation, but is more consistent with a chronic inflammatory pattern accompanied by tissue repair. Previous studies of osteoarticular brucellosis have shown that Brucella infection can involve the spine and joints and is characterised by a chronic and protracted inflammatory course, with treatment strategies and prognosis differing substantially from those of tuberculosis (27, 28). Therefore, the histological findings of the present study not only support the morphological distinction between STB and BS, but also suggest fundamental differences in local host immune responses between the two diseases.

Pathogen detection remains the diagnostic gold standard for both STB and BS. Histopathology should therefore be interpreted as an adjunctive rather than replacement modality, particularly in patients with delayed culture results, low pathogen burden, or prior antimicrobial exposure. In our cohort, the tuberculous nodule-caseous necrosis-Langhans giant cell pattern in STB and the plasma cell infiltration-foamy cell reaction-fibrous tissue proliferation pattern in BS served as lesion-level pathological clues, not definitive stand-alone diagnostic rules.

At the histopathological level, foamy cell reaction was more frequent in BS than in STB, but foamy cells and lipid droplets are not exclusive to BS. Prior studies have described lipid-laden macrophages in pulmonary tuberculosis lesions and have also implicated lipid-remodelled macrophage biology in Brucella-related infection contexts (11, 17). However, most available evidence comes from lung tissue, peripheral immune compartments, or experimental infection models, whereas direct comparative evidence from spinal infectious lesions remains limited. We therefore do not interpret foamy cell reaction as an independent diagnostic cutoff, but we consider its higher frequency in BS lesions, when combined with plasma cell infiltration, fibrous tissue proliferation, pathogen evidence, and scRNA-seq-defined lipid-remodelled macrophage states, to have meaningful adjunctive value for lesion-level pathological differentiation.

The novel aspect of the present study is the direct integration of lesion histopathology and scRNA-seq across two clinically confusable spinal infections that are highly relevant in northwestern China. This design allowed us to relate conventional morphological differences to specific myeloid states and to generate tissue-based evidence that may improve differential pathological interpretation when routine microbiological confirmation is delayed or incomplete. Prior pulmonary tuberculosis single-cell studies have likewise shown heterogeneous macrophage lineages within infected lungs and necrotizing granulomas (11, 17, 30), but cross-tissue comparisons should be interpreted cautiously because lung and spinal lesions represent different anatomical microenvironments. We are therefore treating published pulmonary tuberculosis datasets and spinal brucellar spondylitis datasets as important external context and are conducting further cross-dataset integration as follow-up work rather than presenting it as completed validation in the current manuscript.

A key single-cell finding of this study is that Macro4 was relatively enriched in BS and displayed coordinated lipid-metabolic and tissue-remodelling features. Macro4 highly expressed PPARG, APOE, APOC1, PLIN2, and CYP27A1 on feature plots (**Figure 6B**), and BS_Macro4 showed stronger cholesterol-efflux-related activity than STB_Macro4 in the within-subset comparison (**Figure 6D**). **Figure 6D** should therefore be viewed as one supportive within-subset functional comparison rather than the sole basis for identifying Macro4. The interpretation of Macro4 is instead derived from convergent evidence, including relative enrichment, marker expression, functional module scores, GSVA pathway enrichment, and tissue-level dual immunofluorescence. These convergent features support the interpretation of Macro4 as a lipid-remodelled macrophage state rather than a universally highest-scoring generic foam-cell subset.

M1/M2 scoring showed that Macro4 carried relatively high M2-related signals while retaining some M1-related features, indicating that it cannot be reduced to a simple M1/M2 dichotomy. This mixed profile is more consistent with a chronic infection-associated macrophage state shaped by metabolic stress, tissue repair, and persistent inflammatory signalling.

Transcription-factor analysis showed activation of MITF/TFEC, CEBPD, AP-1 family members, and IRF/NFKB-related regulators in BS-derived macrophages. Brucella abortus has been reported to modulate macrophage polarization and inflammatory responses through NF-kB-related pathways (29), supporting the possibility that the Brucella infection background contributes to immunometabolic remodelling in BS-associated macrophages.

Beyond metabolic remodelling, CellChat analysis suggested that Macro4 may participate in lesion microenvironmental interactions through candidate HGF-MET signalling. Both STB and BS showed complex intercellular communication networks, but the relative contribution of Macro4 to the HGF context was greater in BS. These inferred interactions offer a mechanistic hypothesis linking lipid-remodelled macrophages with endothelial and osteoclast compartments rather than a validated therapeutic target at this stage, and they are biologically plausible in light of prior work on MET receptor activation and macrophage involvement in bone remodelling (24, 25).

Importantly, Macro4 exhibited communication activity in both STB and BS. Therefore, its communication function should not be interpreted as being exclusive to BS. A more reasonable interpretation is that Macro4 participates in lesion microenvironmental regulation in both diseases, but its lipid-remodelled state and HGF-related signalling output in BS are more closely coupled with a chronic inflammation–tissue repair pathological background.

Overall, our data support distinct local immunopathological programs in STB and BS. The main biological advance is the identification of a BS-associated lipid-remodelled macrophage state linked to the foamy cell reaction and to candidate HGF-MET communication, rather than the establishment of a stand-alone diagnostic assay or fully validated therapeutic target.

This study has several limitations. First, although the histopathological cohort was relatively large, only six lesion samples underwent scRNA-seq. Second, adjacent uninvolved tissue was not routinely collected in this surgical debridement cohort. Third, the HGF-MET communication results are transcriptome-inferred and remain hypothesis-generating. Fourth, tissue-level validation in this study is limited to dual immunofluorescence, and broader orthogonal validation such as qPCR, flow cytometry, additional immunohistochemistry, spatial analysis, or functional perturbation remains necessary. In addition, tuberculosis and brucellosis are important infectious diseases requiring strict biosafety control, and viable-pathogen infection experiments, particularly those involving Mycobacterium tuberculosis, require BSL-3 experimental conditions that are not yet fully available locally in our relatively underdeveloped northwestern region. Mechanistic infection experiments therefore could not be completed in the present revision.

## 5 Conclusion

At the histopathological and single-cell transcriptomic levels, STB and BS represent distinct local immunopathological programs. STB is dominated by a granulomatous-necrotizing inflammatory pattern, whereas BS more often shows a chronic inflammatory and tissue-repair-associated lesion background with a more prominent foamy cell reaction. Macro4 is best interpreted as a BS-associated lipid-remodelled macrophage state supported by convergent lesion pathology, scRNA-seq, and tissue-level immunofluorescence findings. These observations provide a framework for future validation-oriented work on differential pathology and host-microenvironment remodelling in infectious spinal disease.

## Data Availability

The raw sequencing data generated in this study have been deposited in the Genome Sequence Archive for Human (GSA-Human) under accession numbers HRA017497 (single-cell RNA-seq) and HRA017547 (bulk RNA-seq). Due to ethical and privacy considerations associated with human sequencing data, the datasets are available through controlled access rather than unrestricted public access. Qualified researchers may apply for access through the GSA-Human repository or contact the corresponding authors with a reasonable request, subject to applicable ethical, privacy, and data-use requirements.
