# Correction: Integrated histopathology and single-cell transcriptomics reveal distinct pathological features and foamy macrophage characteristics in spinal tuberculosis and brucellar spondylitis

**DOI:** 10.3389/fimmu.2026.1985571

**Published:** 2026-09-14

**Authors:** Qiang Liu, Hanhua Guo, Junxiao Liu, Zhengyong Tao, Long Ma, Zhangui Gu, Xuefeng Yue, Daihao Wei, Ningkui Niu, Jiandang Shi, Zongqiang Yang

**Affiliations:** 1 Department of Orthopedic, General Hospital of Ningxia Medical University, Yinchuan, Ningxia Hui Autonomous Region, China; 2 The First Clinical College of Ningxia Medical University, Yinchuan, Ningxia Hui Autonomous Region, China; 3 Research Center for Prevention and Control of Bone and Joint Tuberculosis, General Hospital of Ningxia Medical University, Ningxia, China

**Keywords:** brucellar spondylitis, cell-cell communication, foam cell, lipid metabolism, macrophage, single-cell RNA sequencing, spinal tuberculosis

Author Zongqiang Yang was inadvertently omitted from the author list and corresponding-author information in the published article.

The correct author list and corresponding-author information are provided below:

Qiang Liu1,2†, Hanhua Guo1,2†, Junxiao Liu1,2†, Zhengyong Tao1,2, Long Ma1,2, Zhangui Gu1,2, Xuefeng Yue2, Daihao Wei1, Ningkui Niu1,3*, Jiandang Shi1,3* and Zongqiang Yang1,2,3*

1. Department of Orthopedic, General Hospital of Ningxia Medical University, Yinchuan, Ningxia Hui Autonomous Region, China 2. The First Clinical College of Ningxia Medical University, Yinchuan, Ningxia Hui Autonomous Region, China 3. Research Center for Prevention and Control of Bone and Joint Tuberculosis, General Hospital of Ningxia Medical University, Ningxia, China

The **Author contributions statement** has been corrected to read:

“QL: Methodology, Resources, Software, Supervision, Writing – original draft, Writing – review & editing. HG: Data curation, Project administration, Resources, Software, Writing – original draft, Writing – review & editing. JL: Data curation, Formal analysis, Methodology, Writing – review & editing. ZT: Writing – review & editing, Writing – original draft, Supervision, Data curation, Investigation. LM: Resources, Writing – review & editing. ZG: Investigation, Methodology, Project administration, Writing – review & editing. XY: Formal analysis, Project administration, Software, Writing – review & editing. DW: Software, Visualization, Writing – original draft. NN: Data curation, Investigation, Methodology, Software, Supervision, Writing – original draft. JS: Conceptualization, Data curation, Formal analysis, Funding acquisition, Investigation, Supervision, Writing – review & editing. ZY: Data curation, Funding acquisition, Investigation, Supervision, Writing – original draft, Writing – review & editing.”

The original version of this article has been updated.

